# Ghost guns: spookier than you think they are

**DOI:** 10.1186/s40621-021-00306-0

**Published:** 2021-04-05

**Authors:** Garen J. Wintemute

**Affiliations:** Baker–Teret Chair in Violence Prevention and Professor of Emergency Medicine, University of California, Davis, USA

**Keywords:** Firearms, Violence, Terrorism, Extremism, Political violence, Ghost guns

## Abstract

Off-the-books, untraceable “ghost guns” can now be manufactured at home, easily, and in large numbers; they contribute ever more frequently to firearm violence, including hate violence and domestic terrorism. The Bureau of Alcohol, Tobacco, Firearms and Explosives estimates that in 2019 alone, law enforcement agencies recovered more than 10,000 ghost guns. The manuscript describes the current situation and suggests specific actions that state and federal governments can take to avert disaster.

Suppose I wanted to produce rifles and pistols in large numbers, clandestinely, to arm violent extremists or a hate group in the United States: Donald Trump’s erstwhile shock troops the Proud Boys, for example, members of the Three Percenters or boogaloo movement, or any of scores of private so-called militias and neo-Nazi organizations. You might think this would require a substantial investment and high-level technological expertise on my part. Far from it.

For $2100 I can buy a GhostGunner3—a computer-controlled milling machine not much bigger than a desktop laser printer—that will let me “manufacture firearms with confidence and ease, in the privacy of [my] own home.” (Ghost Gunner 3 Deposit, 2020) The machine produces finished lower receivers for AR-type rifles or frames for Glock-type pistols at the rate of 1 every 35 min, and tooling for AK-type rifles is in development. I can easily download the controlling code, my laptop computer is more than adequate, and helpful chatrooms provide advice.

Finished lower receivers and frames are regulated as firearms under federal law; they are the keystone components to which manufacturers attach the other parts needed to produce fully functional weapons. But nearly-finished aluminum or polymer receiver blanks, as the Bureau of Alcohol, Tobacco, Firearms and Explosives (ATF) calls them—"80-percenters,” as they are known colloquially—are considered nothing more than pieces of metal or plastic. I can buy them for as little as $50 to $75 each, and I can get volume discounts.

If I don’t have the money to buy a milling machine, I can rent one, or I can buy inexpensive jigs and produce finished receivers with a drill press and hand tools. All the other parts that get me to a durable working firearm are readily available. Home manufacturing of firearms for personal use is legal under federal law and requires no license, and supplying parts to home builders and customizers is a growth area for the firearm industry.

Here’s the key: Because I make them myself, these firearms are not required to have serial numbers or other identifiers, which makes it impossible to trace transfers of ownership. Because I want to cover my tracks, I keep no records of their existence. They are guns without a history, coming from nowhere. Ghost guns.

Unserialized firearms, as they’re properly known, are not a new phenomenon. For decades, firearm manufacturers (from large corporations to single individuals) have sought clarification from ATF on the question, When in its production process does a piece of raw material become a firearm in the eyes of the law? There has never been a clear answer; ATF rules on a case by case basis. Originally, the agency held that a substantial amount of effort—work that took ATF’s own experts more than an hour to accomplish—had to be left to the buyer for a partly-finished receiver or frame not to be ruled a firearm. Beginning about 15 years ago, however, ATF not only lowered the bar but provided specific, written instructions on how to come as close as possible to it’s-a-firearm status without crossing the line (Fig 1a).

**Fig. 1:**
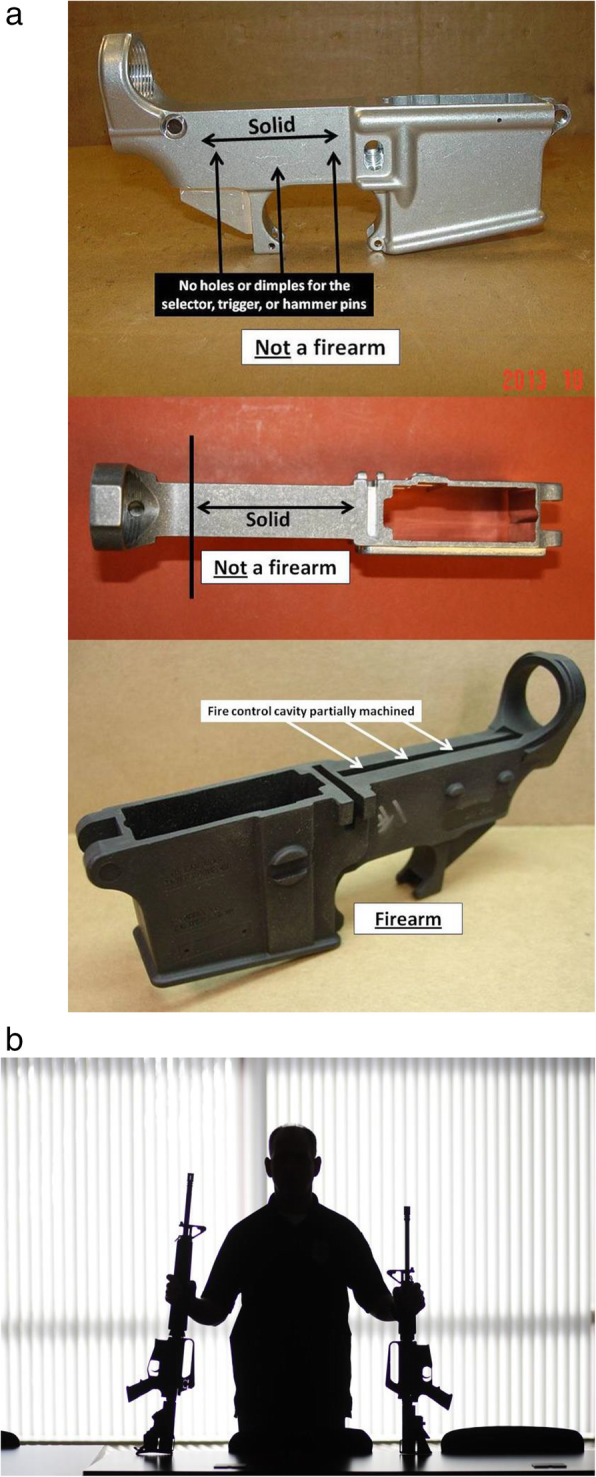
Panel **a**: Finished and unfinished lower receivers for AR 15-type rifles. (Available from https://www.atf.gov/firearms/qa/are-%E2%80%9C80%E2%80%9D-or-%E2%80%9Cunfinished%E2%80%9D-receivers-illegal. Accessed November 19, 2020.) Panel **b**: Unserialized AR-type rifles on display by ATF, 2017. (Available from: https://www.latimes.com/opinion/story/2019-12-22/ghost-guns-atf-regulations-trump-nra. Accessed November 18, 2020. Reprinted by permission.)

There is no estimate of the number of fully functional ghost guns in circulation, though a Colorado researcher estimates that hundreds of thousands of unfinished receivers have been sold (Caron, 2017). This past December, ATF disclosed that law enforcement agencies recovered some 10,000 ghost guns in 2019 (United States District Court, District of Nevada, 2020) (Fig 1b). To put this in perspective, ATF successfully traced nearly 270,000 firearms that year. (Bureau of Alcohol, Tobacco, Firearms and Explosives, 2020). CBS 60 Minutes reported in May 2020 that 38 states had seen criminal cases involving ghost guns, and ATF reported that 30% of all firearms they recovered in California trafficking investigations were unserialized. A Florida man has been convicted of making more than 200 ghost guns, mostly AR-15 type rifles. There have been at least 3 mass shootings with ghost guns in California alone.

The potential for large-scale, clandestine firearm manufacture in support of armed extremist groups is cause for great concern. In October 2020, the Department of Homeland Security (DHS) singled out “domestic violent extremists [as] presenting the most persistent and lethal threat” of terrorist violence in the United States (United States Department of Homeland Security, 2020). DHS’s acting director was “particularly concerned about white supremacist violent extremists, who have been exceptionally lethal in their abhorrent, targeted attacks in recent years.” (United States Department of Homeland Security, 2020)

In mid-2020, an adherent of the boogaloo movement—an active-duty Air Force sergeant—was charged with the murder of 2 law enforcement officers and the attempted murder of a third. “They came to Oakland to kill cops,” the FBI’s lead investigator told the press (Dolan et al., 2020). At least 1 fatality, and possibly all 3 shootings, involved a ghost machine gun. In October, more than a dozen men were arrested for plotting to kidnap Michigan governor Gretchen Whitmer. They were participants in the boogaloo and militia movements, and they had ghost guns.

The number, size, and activity of violent extremist organizations will likely grow now that a new administration, one they rightly view as hostile to their objectives, has taken office. These organizations are fully capable of influencing the course of American public life and have precisely that in mind. The ability to produce their own undocumented firearms or acquire them readily from others increases the threat they pose to the nation's health and security.

Demand for unfinished receivers and milling machines is high and growing, with many sources reporting that they are out of stock, but the ghost is not yet wholly out of the bottle. A relatively small number of important point sources produce unfinished receivers, though many outlets sell them. Defense Distributed, in Texas, may be the only primary source for the milling machines. (That name may ring a bell; several years ago, Defense Distributed developed and promoted technology for 3-D printing of firearms.)

## Actions to be taken

The following specific legislative reforms or administrative actions, if taken soon, would reduce the risk of disaster; 5 states have already taken preliminary steps. Federal and state governments should prohibit unlicensed manufacture of firearms. ATF should establish a clear point, early in the production process, at which unfinished receivers or frames are defined as firearm precursors and further finishing work is defined as manufacturing firearms. Firearm precursors must be given unique, permanently-affixed serial numbers, and individual production and disposition records must be kept. The purchase restrictions now in place for fully functional firearms must be applied to firearm precursors. Owners of the existing stock of ghost guns and unserialized firearm precursors should be required to declare their existence and receive, affix, and record unique serial numbers. After an appropriate delay for implementation, possession of an unserialized firearm or precursor should be a crime for which a conviction prohibits further ownership or acquisition of firearms, precursors, and ammunition.

The firearm industry operates under a regulatory structure that was largely set in place more than 50 years ago. Time and technology have moved on. In February 2020, a high-ranking DHS official described domestic terrorism this way in testimony before Congress: “it feels like we are at the doorstep of another 9/11 …we can see it building and we don’t quite know how to stop it.” (Neumann, 2020)

Americans learned 20 years ago, as we are learning now with COVID-19, of the terrible penalty that can follow failure to recognize and respond adequately to an emerging threat to the public’s health and safety. Ghost guns pose such a threat, which extends beyond political violence to criminal violence of all types—produced clandestinely and untraceable, ghost guns are tailor made for the criminal gun market. Now is the time to do something about them.

## Data Availability

NA
